# Addressing the unique needs of adolescent mothers in the fight against HIV

**DOI:** 10.1002/jia2.25155

**Published:** 2018-06-29

**Authors:** Allison K Groves, Suzanne Maman, Petra H Stankard, Luwam T Gebrekristos, Joseph J Amon, Dhayendre Moodley

**Affiliations:** ^1^ Department of Community Health and Prevention Drexel University Philadelphia PA USA; ^2^ Department of Health Behavior University of North Carolina at Chapel Hills Chapel Hill NC USA; ^3^ Independent Researcher Vienna Austria; ^4^ Woodrow Wilson School of Public and International Affairs Princeton University Princeton NJ USA; ^5^ Department of Obstetrics and Gynaecology University of KwaZulu‐Natal Nelson Mandela School of Medicine Durban South Africa

**Keywords:** adolescent, mothers, prevention, pregnancy, postpartum, sub‐Saharan Africa

## 1 Introduction

Despite increased awareness of and investment in the reduction of HIV among adolescent girls and young women (AGYW) in sub‐Saharan Africa, the unique needs of adolescent mothers remain unaddressed. Teenage birth rates in the region are higher than anywhere else in the world (with a range from 20% to >50%) 1. Furthermore, teenage pregnancy rates over the past decade have risen, not fallen 2, indicating continued unmet reproductive health needs of AGYW in the region.

The perinatal period is a time of increased risk of HIV infection. A recent study found that compared to non‐pregnant women, pregnant women were more than two times more likely to be infected with HIV in pregnancy. The risk continues after childbirth, when postpartum women were found to be four times more likely to be infected with HIV than non‐pregnant women 3. Furthermore, in a study of HIV incidence postpartum, younger women were at higher risk of HIV than older women: incidence declined with each additional year of age 4.

In addition, adolescent mothers are at higher risk of HIV than their non‐parenting counterparts**.** In a longitudinal study, early adolescent pregnancy was associated with a three‐fold HIV risk 5. In another study, adolescents who had ever been pregnant were more likely to report unprotected sex in the last 3 months when compared to nulliparous adolescents (39.8% vs. 30.5%). Furthermore, adolescents who had ever been pregnant were also more likely to report physical partner violence (47.2% vs. 16.8%) and transactional sex (21.9% vs. 16.9%) 6, known relational and structural risk factors for HIV infection 7, 8. Research is needed to understand how the psychological, relational and structural changes that accompany the transition to adolescent motherhood influence unprotected sex.

A recent commentary, published in this journal, highlights how existing PMTCT interventions do not sufficiently address the needs of HIV‐positive adolescent mothers 9. Numerous studies have reported poor retention rates in PMTCT programmes for younger HIV‐positive mothers as compared to older mothers 10, 11, 12, and higher rates of MTCT have been observed among HIV‐exposed infants of younger mothers than among adult mothers 13.

While there is more work to be done to develop appropriate care and treatment models for HIV‐positive adolescent mothers, we cannot overlook the needs of HIV‐negative adolescent mothers to address their very high risk for infection. Global initiatives to address the vulnerability of AGYW have given limited attention to the needs of adolescent mothers. For example, the DREAMS partnership has provided $385 million dollars to deliver interventions that address the structural drivers of HIV infection among AGYW in 10 sub‐Saharan African countries 14, however, few interventions within DREAMS explicitly target adolescent mothers 14. Furthermore, a search of the UNAIDS, WHO, CDC and PEPFAR websites, performed in May 2018, found limited content on HIV prevention needs of adolescent mothers. The four sites were examined for content containing the terms “HIV”, “risk” and at least one of the following: “AGYW,” “adolescent,” “youth,” “young people,” “children,” and “teen”. A total of 61,038 items (not necessarily independent) were retrieved and then reviewed using the terms “adolescent mother,” “young mother,” “teen mother,” or “teenage mother” to identify items describing original research or interventions. A total of four items were found that discussed the rights of teen mothers to go to school (n = 1), the need to reduce risk in teen mothers' sexual relationships (n = 1), the importance of adolescent‐friendly clinical services (n = 1) and the need for employment opportunities (n = 1) 15, 16, 17, 18. Finally, a review of NIH RePORT in May 2018 using the same approach yielded 17,830 funded studies. Only one included a focus on adolescent mothers: an intervention designed to increase the capacity of community health workers to provide HIV prevention and care through home visits in the postpartum period. Similarly, in this study, adolescent mothers were only one sub‐population of interest in the larger study population 19.

Pregnant adolescents initiate antenatal care later and are less likely to test for HIV than adult women, and pregnancy‐related stigma and poor quality of care contribute to these behaviours 20. However, there is no research on HIV‐negative adolescent girls' experiences with or receptivity to post‐test counselling within the context of PMTCT programmes. Furthermore, we know nothing about adolescent mothers' trajectories of sexual behaviour, even though many young women's relationships with their partners change substantially during the perinatal period 21, 22.

Our gap in understanding the needs of HIV‐negative adolescent mothers extends postpartum. Adolescent mothers are susceptible to postpartum depression 23, yet there is a paucity of data on how poor mental health postpartum impacts HIV risk for this sub‐population and what the best avenues for intervention may be. Furthermore, adolescent mothers face significant challenges to returning to school 24 and school dropout is associated with increased risk of HIV infection 25.

In sum, to develop effective HIV prevention interventions for adolescent mothers, we need a cohesive research agenda to understand the multilevel mechanisms that increase their risk of HIV following childbirth. First, research should determine whether there are biological differences between adult and adolescent women that increase younger women's risk of HIV acquisition during the perinatal period. Second, research is needed to understand how the psychological, relational and structural changes that accompany the transition to motherhood influence adolescent mothers' likelihood of engaging in unprotected sex after birth. A comprehensive understanding of the mechanisms that foster biological and psychosocial wellbeing, healthy relationships and positive school outcomes will inform development of tailored interventions for adolescent mothers.

There is a growing list of evidence‐based behavioural and biomedical HIV prevention modalities including the use of pre‐exposure prophylaxis. It is also evident that no single intervention on its own can produce the desired effects of HIV prevention, and that combination intervention strategies should be tailored to address the unique needs of target groups 26, 27. Interventions that have targeted individual and structural determinants of HIV risk in other populations might be adapted and evaluated for HIV‐negative adolescent mothers. For example, psychosocial interventions could target adolescent mother's interpersonal and behavioural skills 28 and in so doing, may reduce postnatal depression 29. Furthermore, structural interventions, like cash transfers 30, may decrease the economic costs of childbearing and subsequently facilitate adolescent mother's return to school. Finally, increasing the accessibility of adolescent‐friendly health clinics 31 for prenatal and postnatal care may increase uptake of services and engagement in care for adolescent mothers.

Adolescent motherhood is more common in sub‐Saharan Africa than anywhere else in the world and occurs against a backdrop of the world's highest HIV rates. Despite this, young mothers, and especially HIV‐negative adolescent mothers, have garnered limited attention as a distinctly vulnerable group. Research and interventions that seek to understand and account for the experience of adolescent motherhood are critical for the health of adolescent mothers, the health of their children, and the health of the continent.

## Competing interests

There are no conflicts of interest to report.

## Authors' contributions

AKG conceptualized and drafted the commentary. SM, PHS, JJA and DM provided substantial feedback on the draft and subsequent revisions. LTG led the content analysis and also provided feedback on the draft and subsequent revisions.

## References

[1] Adolescent pregnancy [Internet]. 2018. [cited 2018 Apr 30]. Available from: http://www.who.int/en/news-room/fact-sheets/detail/adolescent-pregnancy

[2] Population Fund . Motherhood in childhood: facing the adolescent pregnancy. New York, NY: UNFPA; 2013 116 p. (State of world population).

[3] Thomson KA , Hughes J , Baeten JM , John‐Stewart G , Celum C , Cohen CR , et al. Increased risk of female HIV‐1 acquisition throughout pregnancy and postpartum: a prospective per‐coital act analysis among women with HIV‐1 infected partners. J Infect Dis. 2018;218(1):16–25. 29514254 10.1093/infdis/jiy113 PMC5989601

[4] Humphrey JH , Hargrove JW , Malaba LC , Iliff PJ , Moulton LH , Mutasa K , et al. HIV incidence among post‐partum women in Zimbabwe: risk factors and the effect of vitamin A supplementation. AIDS. 2006;20(10):1437–46. 16791019 10.1097/01.aids.0000233578.72091.09

[5] Christofides NJ , Jewkes RK , Dunkle KL , Nduna M , Shai NJ , Sterk C . Early adolescent pregnancy increases risk of incident HIV infection in the Eastern Cape, South Africa: a longitudinal study. J Int AIDS Soc. 2014;17(1):18585. 24650763 10.7448/IAS.17.1.18585 PMC3962027

[6] Stoner MC , Edwards JK , Rucinski K , Selin A , Hughes JP , Wang J , et al. The relationship between school dropout and pregnancy among young women in South Africa: a HPTN 068 analysis. J Adolesc Health. Review. 10.1177/1090198119831755 PMC6625926 30819011

[7] Li Y , Marshall CM , Rees HC , Nunez A , Ezeanolue EE , Ehiri JE . Intimate partner violence and HIV infection among women: a systematic review and meta‐analysis. J Int AIDS Soc. 2014;17(1):18845. 24560342 10.7448/IAS.17.1.18845 PMC3925800

[8] Wamoyi J , Stobeanau K , Bobrova N , Abramsky T , Watts C . Transactional sex and risk for HIV infection in sub‐Saharan Africa: a systematic review and meta‐analysis. J Int AIDS Soc. 2016;19(1):20992. 27809960 10.7448/IAS.19.1.20992 PMC5095351

[9] Callahan T , Modi S , Swanson J , Ng'eno B , Broyles LN . Pregnant adolescents living with HIV: what we know, what we need to know, where we need to go. J Int AIDS Soc. 2017;20(1):21858. 28782334 10.7448/IAS.20.1.21858 PMC5577684

[10] Kirsten I , Sewangi J , Kunz A , Dugange F , Ziske J , Jordan‐Harder B , et al. Adherence to combination prophylaxis for prevention of mother‐to‐child‐transmission of HIV in Tanzania. PLoS ONE. 2011;6(6):e21020. 21695214 10.1371/journal.pone.0021020 PMC3112206

[11] Ronen K , McGrath CJ , Langat AC , Kinuthia J , Omolo D , Singa B , et al. Gaps in adolescent engagement in antenatal care and prevention of mother‐to‐child HIV transmission services in Kenya. JAIDS J Acquir Immune Defic Syndr. 2017;74(1):30–7. 27599005 10.1097/QAI.0000000000001176 PMC5895459

[12] Horwood C , Butler LM , Haskins L , Phakathi S , Rollins N . HIV‐infected adolescent mothers and their infants: low coverage of HIV services and high risk of HIV transmission in KwaZulu‐Natal, South Africa. PLoS ONE. 2013;8(9):e74568. 24073215 10.1371/journal.pone.0074568 PMC3779214

[13] Woldesenbet SA , Jackson D , Goga AE , Crowley S , Doherty T , Mogashoa MM , et al. Missed opportunities for early infant HIV diagnosis: results of a national study in South Africa. J Acquir Immune Defic Syndr. 2015;68(3):e26. 25469521 10.1097/QAI.0000000000000460 PMC4337585

[14] DREAMS .DREAMS Core Package of Interventions Summary [Internet]. [cited 2018 May 15]. Available from: https://www.pepfar.gov/documents/organization/269309.pdf

[15] Women UN, UNICEF . International technical guidance on sexuality education: an evidence‐informed approach. Paris, France: UNESCO Publishing; 2018.

[16] UNAIDS . ALL IN to end the adolescent AIDS epidemic [Internet]. 2016 Available from: http://www.unaids.org/sites/default/files/media_asset/ALLIN2016ProgressReport_en.pdf

[17] South African National AIDS Council . Let our actions count: South Africa's national strategic plan for HIV, TB and STIs 2017–2022 [Internet]. South African National AIDS Council Pretoria; 2017 Available from: http://sanac.org.za/2018/03/06/download-the-full-version-of-the-national-strategic-plan-for-hiv-tb-and-stis-2017-2022/

[18] Swaziland Country Operational Plan (COP). Strategic Direction Summary [Internet]. Pepfar.gov. 2017 [cited 2018 May 15]. Available from: https://www.pepfar.gov/documents/organization/273039.pdf

[19] NIH RePORTER . An RCT to improve the South African government's community health workers' capacities to deliver evidence‐based interventions for optimizing HIV outcomes and reducing its comorbidities [Internet]. 2018 [cited 2018 May 15]. Available from: https://projectreporter.nih.gov/project_info_description.cfm?aid=9350413&icde=39470867&ddparam=&ddvalue=&ddsub=&cr=1&csb=default&cs=ASC&pball=

[20] Varga C , Brookes H . Factors influencing teen mothers' enrollment and participation in prevention of mother‐to‐child HIV transmission services in Limpopo Province. South Africa. Qual Health Res. 2008;18(6):786–802. 18503020 10.1177/1049732308318449

[21] Hill LM , Maman S , Groves AK , Moodley D . Social support among HIV‐positive and HIV‐negative adolescents in Umlazi, South Africa: changes in family and partner relationships during pregnancy and the postpartum period. BMC Pregnancy Childbirth. 2015;15(1):117. 25982187 10.1186/s12884-015-0542-z PMC4437750

[22] Madhavan S , Harrison A , Sennott C . Management of non‐marital fertility in two South African communities. Cult Health Sex. 2013;15(5):614–28. 23600721 10.1080/13691058.2013.777475 PMC3674186

[23] Yozwiak JA . Postpartum depression and adolescent mothers: a review of assessment and treatment approaches. J Pediatr Adolesc Gynecol. 2010;23(3):172–8. 20496498 10.1016/j.jpag.2009.09.003

[24] Grant MJ , Hallman KK . Pregnancy‐related school dropout and prior school performance in KwaZulu‐Natal. South Africa. Stud Fam Plann. 2008;39(4):369–82. 19248721 10.1111/j.1728-4465.2008.00181.x

[25] Stoner MC , Pettifor A , Edwards JK , Aiello AE , Halpern CT , Julien A , et al. The effect of school attendance and school dropout on incident HIV and HSV‐2 among young women in rural South Africa enrolled in HPTN 068. Aids. 2017;31(15):2127–34. 28692544 10.1097/QAD.0000000000001584 PMC5599334

[26] Anderson S‐J , Cherutich P , Kilonzo N , Cremin I , Fecht D , Kimanga D , et al. Maximising the effect of combination HIV prevention through prioritisation of the people and places in greatest need: a modelling study. The Lancet. 2014;384(9939):249–56. 10.1016/S0140-6736(14)61053-9 25042235

[27] Pettifor A , Nguyen NL , Celum C , Cowan FM , Go V , Hightow‐Weidman L . Tailored combination prevention packages and PrEP for young key populations. J Int AIDS Soc. 2015;18 2S1:1. 10.7448/IAS.18.2.19434 PMC4344537 25724507

[28] Stockings EA , Degenhardt L , Dobbins T , Lee YY , Erskine HE , Whiteford HA , et al. Preventing depression and anxiety in young people: a review of the joint efficacy of universal, selective and indicated prevention. Psychol Med. 2016;46(1):11–26. 26315536 10.1017/S0033291715001725

[29] Kleiber BV , Dimidjian S . Postpartum depression among adolescent mothers: a comprehensive review of prevalence, course, correlates, consequences, and interventions. Clin Psychol Sci Pract. 2014;21(1):48–66.

[30] Owusu‐Addo E , Renzaho A , Smith BJ . The impact of cash transfers on social determinants of health and health inequalities in sub‐Saharan Africa: a systematic review. Health Policy Plan. 2018;33(5):675–96. 29762708 10.1093/heapol/czy020 PMC5951115

[31] Denno DM , Hoopes AJ , Chandra‐Mouli V . Effective strategies to provide adolescent sexual and reproductive health services and to increase demand and community support. J Adolesc Health. 2015;56(1):S22–41. 25528977 10.1016/j.jadohealth.2014.09.012

